# Associations Between Mental Health Conditions and Falls in Older Adults: An Umbrella Review of Systematic Reviews

**DOI:** 10.1093/geroni/igaf122.1756

**Published:** 2025-12-31

**Authors:** Mahederemariam Dagne, Shelly Aboagye, Elizabeth Terhune, Erin Staker, Malaz Hassan, Samia Jones, Aderonke Aderonmu, Anita Rizvi

**Affiliations:** Marymount University, Arlington, Virginia, United States; Marymount University, Arlington, Virginia, United States; Marymount University, Arlington, Virginia, United States; Center for Optimal Aging, Arlington, Virginia, United States; Center for Optimal Aging, Arlington, Virginia, United States; Center for Optimal Aging, Arlington, Virginia, United States; Center for Optimal Aging, Arlington, Virginia, United States; University of Ottawa, Ottawa, Ontario, Canada

## Abstract

Falls are a leading cause of injury and reduced quality of life in older adults, with evidence suggesting a bidirectional relationship between falls and mental health conditions. Depression, anxiety, and psychosocial factors may influence fall risk and outcomes, yet findings across studies remain inconsistent. This umbrella review synthesizes evidence from systematic reviews to clarify these associations and identify potential moderating and mediating factors. We conducted a comprehensive search across MEDLINE, EMBASE, Web of Science, CINAHL, PsycINFO, LILACS, Cochrane, and KSR Evidence. Eligible reviews examined relationships between mental health conditions (e.g., depression, anxiety) and falls in adults aged ≥60 years. Dual independent screening and data extraction will be performed, with quality assessment using AMSTAR 2. Approximately 1780 articles were obtained from MeSH keyword searches. Preliminary findings suggest a strong association between depression and increased fall risk, with psychosocial factors, including loneliness, as potential moderators. Variability in mental health assessment tools and fall reporting methods contributed to heterogeneity in results. This review will synthesize the current body of evidence on the association between mental health and falls, assess methodological quality, and highlight gaps for future research. The findings will inform clinical guidelines and intervention strategies to improve fall prevention and mental health management in aging populations.

